# Medical Journals — Should the Young Practitioner Read Them?

**Published:** 1856-03

**Authors:** 


*Medical Journals — Should the Young Practitioner Read them?* — A well regulated medical periodical is the medium of communication of the experience, thoughts, and reflections of medical men to one another, and of the important discoveries in the different departments of medicine. They supply on a large scale, in a certain degree, the place of societies and associations for improvement, by affording the members of a profession, widely-scattered, a medium for the rapid interchange of views, and for the discussion of principles and methods of practice. While activity in the cultivation of the various branches of medicine is necessary to their healthy existence, they reciprocally tend to stimulate the growth and development of these branches, by the wide diffusion of recent discoveries among earnest laborers in these fields of research.

A serial publication thus becomes, not only a record of the progress of medical science, but also a great repository of facts, valuable for future study. It is the inexhaustible store-house from which the compilers of practical treatises obtain the materials for their works.

The high-toned, liberal, and independent medical periodical is essential, also, to the ethics of the profession. It creates and maintains a sentiment adverse to the petty jealousies and envious strifes, which too often arise between individual members, and totally subversive to all the low arts by which the charlatan imposes upon public credulity.

But the laborious country practitioner can best appreciate the well-regulated periodical. Isolated from all professional associations, he is deprived of the satisfaction of communicating his doubts and perplexities to others, and from mutual discussion deriving light and assistance. His position affords him but limited advantages and time for study and careful investigation; while, from the instructive field of pathology he is entirely debarred. To this large and most respectable class, the medical press is a desideratum. It brings them in close communication with their brethren, scattered over the civilized world, and makes them participants of whatever learning, research, or experience can afford. It spreads before them, for contemplation, the precepts of the public teachers of their profession the discoveries of the microscopist and pathologist, and the experience of the hospital physician and surgeon.

We are led to these remarks by the annually repeated advice of a prominent medical teacher, to his graduating class, never to read medical periodicals. "If you have leisure," he is accustomed to say, with characteristic emphasis, "and must read, select novels instead of medical journals, for your instruction."

We can best prove the injustice of this slur upon medical journals, and show its ill effects upon the young practitioner who has the simplicity to follow it, by making an extract from a letter just received from a young physician, located in the interior of an adjoining state. He says:

"I settled in this place about two years since, under the shadow of an old physician, who had long monopolized the practice of the town. During the first year and a half I had nothing to do; but, undaunted, applied myself diligently to a careful review of my studies, and the perusal of the best medical periodicals. The opportunity finally offered for me to apply my knowledge to good account. I was called to visit a young man with a dislocated femur, which the old physician, my rival, had in vain attempted to reduce with pullies, after torturing the patient several hours, to the horror of the bystanders. Before visiting the patient, I carefully reviewed the admirable, and to me invaluable paper of Dr. Markoe, in the N. Y. Journal of Medicina, on reducing the dislocated femur by manipulation. I found the patient and friends alarmed, and fearful of having instruments again used. I placed him in the proper position for the manipulation, and, in the presence of a multitude of bystanders, began the required movements of the limb. Without causing the slightest pain, I carried it through the proper circle, and was about to bring it down, when the head slipped gently into its socket, to the great relief of the patient, and the satisfaction of the friends. I need hardly add, that within one month of that operation, I had all the business I could do."

We could exhibit many other facts of a similar nature, from our correspondence, illustrating the importance of medical journals to the practitioner, but it would be a supererogatory task. Their value is indisputable to every lover of his profession, and it is to this class we address ourselves.—*N. Y. Journal of Medicine.*

